# The Relationship between Fearfulness, GABA+, and Fear-Related BOLD Responses in the Insula

**DOI:** 10.1371/journal.pone.0120101

**Published:** 2015-03-26

**Authors:** Ilona Lipp, C. John Evans, Caroline Lewis, Kevin Murphy, Richard G. Wise, Xavier Caseras

**Affiliations:** 1 Cardiff University Brain Research Imaging Centre (CUBRIC), School of Psychology, Cardiff University, Cardiff, United Kingdom; 2 MRC Centre for Neuropsychiatric Genetics and Genomics, Institute of Psychological Medicine and Clinical Neurosciences, Cardiff University, Cardiff, United Kingdom; Technion - Israel Institute of Technology, ISRAEL

## Abstract

The inhibitory neurotransmitter GABA plays a crucial role in anxiety and fear, but its relationship to brain activation during fear reactions is not clear. Previous studies suggest that GABA agonists lead to an attenuation of emotion-processing related BOLD signals in the insula. The aim of this study was to investigate the relationship between GABA concentration and fear-related BOLD responses in this region. In 44 female participants with different levels of fearfulness, GABA concentration in the left insula was measured using a GABA+ MRS acquisition during rest; additionally, BOLD signals were obtained during performance of a fear provocation paradigm. Fearfulness was not associated with GABA+ in the left insula, but could predict fear-related BOLD responses in a cluster in the left anterior insula. The BOLD signal change in this cluster did not correlate with GABA+ concentration. However, we found a significant positive correlation between GABA+ concentration and fear-related BOLD responses in a different cluster that included parts of the left insula, amygdala and putamen. Our findings indicate that low insular GABA concentration is not a predisposition for fearfulness, and that several factors influence whether a correlation between GABA and BOLD can be found.

## Introduction

Fear is an acute behavioural and physiological reaction to perceived threat, which has been observed in all mammals and probably evolved because it is useful for survival and avoidance of pain [1]. Recent research using functional magnetic resonance imaging (fMRI) has identified the amygdala and the anterior insula as key brain structures associated with the experience of fear [2–4]. While the amygdala is important for the detection of environmental ‘fear’ cues [5], the anterior insula seems to play the role of integrating internal bodily perceptions and information from external cues to create the experienced emotional state [6]. Both these structures have shown increased levels of activity when phobic participants are presented with phobia related material [7–10], but also when healthy controls are confronted with negative images [7,11,12].

Studies using magnetic resonance spectroscopy (MRS) have also shown that individuals suffering from anxiety disorders have reduced GABA concentration in the occipital cortex [13], the anterior cingulate and basal ganglia [14], and the insula [15]. Also, by enhancing GABA transmission pharmacologically, fear responses [1,16] and emotion related BOLD responses in the insula and the amygdala are attenuated [17–19]. All these suggest a relationship between GABA neurotransmission and fear-related BOLD responses. However, thus far, this hypothesis has not been directly tested. Previous studies have reported a negative relationship between stimulus-induced BOLD contrast and GABA in the visual cortex [20–22] and the anterior cingulate [23]. Our aim was to investigate the relationship between fear induced BOLD responses and GABA concentration in the insula. We recruited participants with either high or low fearfulness and confronted them with a paradigm designed to elicit fear-related BOLD responses. GABA concentration in the insula was assessed in a separate MRS scan at the end of the same scanning session. We expected stronger BOLD responses upon fear inducing stimuli in the insula and amygdala of highly fearful participants, as well as lower GABA concentration in the insula. We also expected a negative correlation between fear-related BOLD changes and GABA concentration in the insula.

## Methods

### 2.1. Participants

Five-hundred and seventy-four females (Mean[Std] age = 21[4]) from Cardiff University (students and staff) underwent an online screening, consisting of the Fear-Survey Schedule-II (FSSII, [24]) and the Fear of Spider Questionnaire (FSQ, [25]). The FSSII consists of 51 items assessing fear to a wide variety of potential stimuli/situations. The FSQ consists of 18 items assessing fear of spiders; this questionnaire has also shown to discriminate among levels of spider fear within non-phobic population [26], which was important for our recruitment strategy. Both questionnaires have previously shown adequate psychometric properties [24–26].

**Fig 1:**
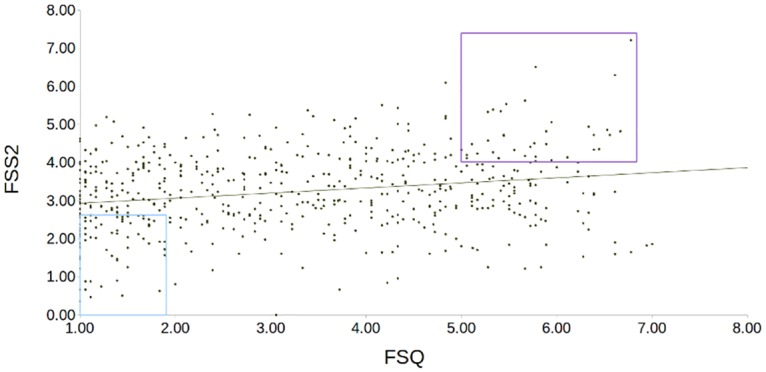
Recruitment of extreme groups. The scatter plot for the whole screening sample (N = 574) is shown, with scores in the Fear of Spider questionnaire on the x-axis, and the Fear Survey Schedule II on the y-axis. The purple box illustrates the recruitment thresholds for the high-fear group, the blue box the thresholds for the low-fear group.

Since our aim was to recruit a sample of participants with either high or low fearfulness and to induce fear in them via the presentation of still images of specific feared stimuli, we invited candidates with the lowest and highest scores in both questionnaires to participate in the imaging study. Therefore, we aimed for a group of low fearful participants who were also not afraid of spiders, and a group of high fearful participants who all shared their fear of spiders. Fig. 1 illustrates the recruitment criteria on both questionnaires.

Candidates were screened over the telephone to ascertain their MRI compatibility, right-handedness, and absence of current or personal history of psychosis, mood or anxiety disorders—other than potential specific spider phobia—according to the MINI International Neuropsychiatric Interview (MINI, [27]). On the day of the scan, participants were requested to complete again the FSSII and FSQ, along with the State and Trait Anxiety Inventory (STAI, [28]), Hospital Anxiety and Depression scale (HADS; [29]), and the General Health Questionnaire (GHQ-12, [30]). Table 1 shows the mean scores on all the questionnaires.

**Table 1:** Questionnaires.

Measure	High fear	Low fear	*t*	*p*
Age	21.5 (3.1)	21.1 (2.9)	0.48	.63
FSQ	5.30 (0.85)	1.22 (0.40)	18.9	< .001
FSS-II	4.19 (0.71)	1.93 (0.80)	9.2	< .001
STAI state	1.60 (0.39)	1.55 (0.30)	0.46	.65
STAI trait	2.03 (0.14)	1.82 (0.11)	1.69	.10
GHQ	0.86 (0.34)	0.79 (0.30)	0.72	.47
HADS anxiety	1.07 (0.33)	0.84 (0.22)	2.46	.02
HADS depression	0.70 (0.30)	0.55 (0.16)	1.92	.06

Questionnaire scores between the two groups are compared (N per group = 19; from each originally recruited group (N = 22), three participants had to be excluded because their scores on the questionnaires did not match their original initial group assignment). Mean (standard deviation) are listed separately for the high fear and the low fear group, the reported *t* and *p* value are obtained from a 2-sample *t-*test. FSQ = Fear of Spider Questionnaire, FSS-II = Fear Survey Schedule—II, STAI = State Trait Anxiety Inventory, GHQ = General Health Questionnaire, HADS = Hospital Anxiety and Depression scale.

We scanned 44 participants, 22 in the high fear group and 22 in the low fear group. Three participants from each group had to be excluded because their scores on the screening questionnaires at the time of scanning did not reflect their original group assignment (their score lay on the other side of the total median). One participant of the low fear group had to be excluded due to problems during the acquisition of the functional imaging data. The final sample consisted of 37 participants, 19 in the high fear group and 18 in the low fear group.

Due to some evidence for an influence of the menstrual cycle on GABA levels [31,32], participants were asked to come for the imaging study during the first 9 days of their cycle; during this period the probability of being in the follicular phase—during which steroid hormone levels are most stable—is 95% [33]. Three participants did not comply with these instructions: one participant in the low fear group came on day 10, and two participants in the high fear group came on day 12 and day 14, respectively. Participants who were taking hormonal contraception (11 in the high fear and 11 in the low fear group) were asked to come for the scanning session outside their pill-free period, if applicable. The study was approved by the Cardiff University School of Psychology Ethics Committee and written informed consent was obtained from all participants. Participants were financially compensated for their time.

### 2.2. Fear inducing paradigm

The fear inducing paradigm involved presenting still pictures of spiders, of other control animals (birds, caterpillars, snails and lizards), generally negative pictures taken from the International Affective Picture System (IAPS, [34]) and neutral pictures also obtained from the IAPS. This allowed us to produce a fear-specific contrast *SPIDERS > ANIMALS* and a fear-unspecific contrast *IAPSnegative > IAPSneutral*. The images were presented in short blocks of 10 seconds, with 4 images (presented for 2.5 sec.) each. After half of the blocks a fixation cross appeared for either 7, 9, 11 or 13 seconds (there were no blocks of the same kind one after the other without a fixation period in between). For each condition, 10 blocks were presented (for more detail see S1 File).

In order to guarantee that participants were processing the images presented and not avoiding the more unpleasant pictures, they were instructed to perform a covert task of responding (button press with right index and middle finger) whether they could detect the presence of a human in the picture (50% of the pictures). The tasks were presented in the scanner using Presentation (Neurobehavioral Systems, Albany, CA) and rear-projected onto a screen behind the participant's head that was visible through a mirror mounted on the RF head coil. After scanning, participants were asked to rate the pictures using a 1 (*very negative*) to 9 (*very positive*) scale based on Lang et al’s [34] pleasure dimension of the Self-Assessment-Manikin scale [35].

### 2.3. Imaging protocol

All data were acquired using a 3T GE HDx MRI System, using a body transmit RF coil and an eight channel receive-only head coil.

#### 2.3.1. Structural scans

A T1 weighted whole-brain structural scan was acquired for purposes of image and MRS voxel registration (3D fast, spoiled gradient echo, TR/TE = 7.9/3.0 ms, TI = 450 ms, Flip angle = 20 deg, 1 x 1 x 1 mm resolution, 256 x 256 x 176 matrix size).

#### 2.3.2. Functional scan: fear inducing paradigm

During task performance, gradient-echo echo-planar T2* images of the entire brain. Forty-six interleaved 2 mm (1 mm gap) AC-PC parallel slices were obtained per volume (204 volumes) with a TR = 3 s, TE = 35 ms, matrix = 64 x 64, FOV = 220 mm, flip angle = 90^o^.

#### 2.3.3. GABA+ magnetic resonance spectroscopy

**Fig 2:**
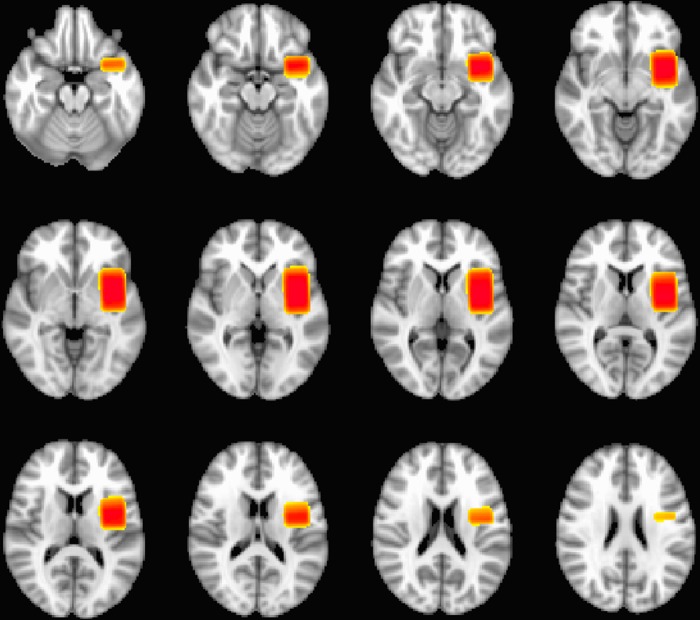
Insula voxel position. This shows the areas that a participant's voxel covered with 85% probability. This map was used for performing the restricted higher-level analysis.

GABA+ was quantified from a 25 x 30 x 40 mm voxel located in the left insula and aligned with the insula cortex in an anterior–posterior direction (see Fig. 2). Two spectra were acquired for each participant. GABA+ data (GABA plus coedited macromolecules) were acquired using a J difference editing technique (MEGA-PRESS, [36]). Spectra were acquired with TR = 1800 ms, TE = 68 ms, 300 transients of 4096 data points were acquired in 9 minutes. Gaussian editing pulses (of 16 ms duration) were applied either to the GABA+ spins (1.9 ppm) or symmetrically about the water peak (7.5 ppm) in an interleaved manner. A further eight transients were acquired, without water suppression, in order to obtain water concentration as an internal concentration reference (GABA/water). For a fuller description of this method please refer to Puts & Edden [37].

All spectra were analysed using Gannet [38]. GABA+ values were corrected for the tissue composition of the voxel as follows: Tissue segmentation was performed using FAST. The water concentration (used as the reference concentration) was corrected for voxel water content according to Ernst et al. [39], as implemented in Gannet. The GABA+ signal was divided by the fraction of tissue in the MR voxel to account for the fact that the GABA concentration in the CSF is negligible (for a similar approach see [40]). Groups did not significantly differ in their voxel gray matter (*t*[35] = 1.05, *p* = .30) or white matter (*t*[35] = -0.11, *p* = .91) content. Prior to using GABA+ values for analysis, all spectra were visually inspected independently by two researchers, and rated using a 3-point scale (2 = *very good*, 1 = *satisfactory*, 0 = *reject*), to ensure the presence of artefacts did not affect the quantification of GABA. Spectra scoring below 1 were rejected, resulting in the exclusion of 26/74 spectra from the dataset. The GABA+ concentration estimations from the two scans per voxel were averaged for each participant if two spectra were available. Altogether, usable GABA+ data was acquired for 29 participants (all included spectra can be seen in S1 Fig.).

### 2.4. Physiological parameters

The following physiological parameters were recorded during the scanning session: a) the cardiac cycle was recorded using a pulse-oximeter placed on the left index finger, b) a respiration trace was recorded with a pneumatic belt around the chest, c) end-tidal carbon dioxide (PetCO_2)_) and end-tidal oxygen (PetO_2)_) were recorded using a nasal cannula attached to rapidly responding gas analysers (AEI Technologies, PA) to provide representative measures of arterial partial pressures of both gases.

### 2.5. Data preprocessing and analyses

The BOLD fMRI time-series data during the emotion paradigm were first corrected for physiological noise. This correction consisted of applying correction of cardiac and respiratory artifacts (RETROICOR, [41])—using two cardiac, two respiratory and one interaction component and of the variance related to carbon dioxide (PetCO_2_) level, oxygen (PetO_2_) level (both HRF convolved), heart rate (HR; CRF convolved; [42]) and respiratory volume per time (RVT; RRF convolved; [43]), using a general linear model framework. Both steps were performed using Matlab (The MathWorks Inc., vs. R2011a). Physiological noise correction was performed prior to analysing the data for task responses. For seven participants at least one of the physiological parameters could not be analysed due to technical difficulties during recording. For these participants physiological noise correction was performed with the remaining parameters, for two participants physiological recordings were missing altogether.

The corrected dataset was subsequently analyzed using FEAT (FMRIB Expert Analysis Tool, v5.98, http://www.fmrib.ox.ac.uk/fsl, Oxford University, UK). Preprocessing steps before model fitting were applied to each participant’s time-series, and included: highpass filtering of the data (100 s temporal cutoff), non-brain removal using BET [44],”MCFLIRT” motion correction [45], spatial smoothing with a Gaussian kernel of full-width-half-maximum 5 mm and fieldmap-based EPI unwarping using PRELUDE + FUGUE [46,47]; for three participants this was not performed due to problems during the acquisition of the fieldmaps. Functional images were registered using FLIRT [48] in a first step to the structural image with 6 degrees of freedom, and in the second step to the Montreal Neurological Institute (MNI) space with 12 degrees of freedom and FNIRT non-linear (10 mm) warp [49,50]. GABA+ measures were not correlated with head motion during the fear provocation task (mean displacement and covariance between task and head motion).

To model the fear provocation task, four event types were defined, one for each picture condition (i.e. *IAPSnegative*, *IAPSneutral*, *SPIDERS*, *ANIMALS*). Fixation cross periods were used as the baseline. The model was convolved with the hemodynamic response function (gamma convolution), and the same temporal filtering was applied to the model as to the data. Temporal derivatives were included as regressors of no interest. Two main contrasts of interest were defined: 1) *SPIDERS > ANIMALS*, 2) *IAPSnegative > IAPSneutral*. Group average and group difference (high fear vs. low fear) maps were created with a mixed effects model using FLAME1. For the analysis looking at the influence of GABA+ on the BOLD responses, the demeaned GABA+ measures were entered as a regressor in the group analysis model. For participants with no GABA+ data, the mean value was entered. The Z (Gaussianised T/F) statistic images were thresholded using clusters determined by Z > 2.3 and a (corrected) cluster significance threshold of P <. 05 [51].

For all correlations we defined bivariate outliers based on the overall structure of the data using the Matlab toolbox provided by Pernet et al. [52], and Pearson correlation coefficients were computed with the remaining data points. Due to outlier removal, sample size changed slightly for each reported correlation. We therefore report correlations with respective degrees of freedom in brackets.

ANOVAs were computed using the Matlab functions anova1 and mixed_between_anova (www.mathworks.co.uk/matlabcentral/fileexchange/27080-mixed-betweenwithin-subjects-anova/content/mixed_between_within_anova.m).

## Results

### 3.1. Behavioural responses

Performance on the covert task was very high with a mean accuracy of 93% (*Min*. = 73%, *Std*. = 6%), suggesting that participants did pay attention to the stimuli. We did not find group differences in accuracy or reaction times (*F*[1,36] = 0.23, *p* = .64), and no interaction between group and stimulus category (*F*[3,108] = 0.36, *p* = .78).

With regard to the picture ratings, we found a significant interaction between group and stimulus category (*F*[3,102] = 6.5, *p* <. 001). This interaction was driven by the group difference in the ratings for spiders (*F*[1,34] = 11.6, *p* <. 01) but not for any of the other categories. Pictures of spiders were rated significantly less pleasant by participants in the high fear group (*Mean* = 2.2, *Std*. = 1.3) as compared to participants in the low fear group (*Mean* = 5.6, *Std*. = 2.9). Spiders were perceived as significantly more negative than the control animals in both of the groups (high fear: *F*[1,17] = 58, *p* <. 01; low fear: *F*[1,17] = 9.08, *p* <. 01), and the negative IAPS pictures as more negative than the neutral pictures (high fear: *F*[1,17] = 67, *p* <. 01; low fear: *F*[1,17] = 40, *p* <. 01).

### 3.2. Group activation and group differences in BOLD and GABA+ signal

**Fig 3:**
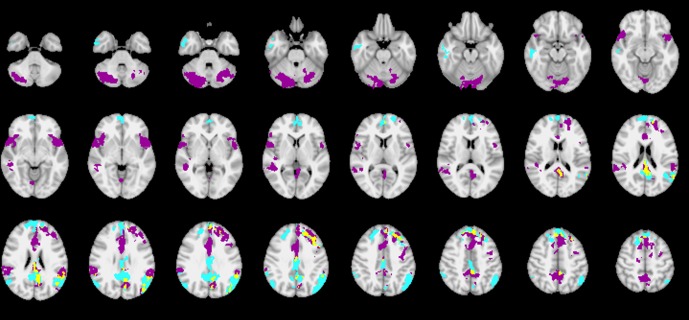
Fear-specific contrast. High fear (N = 19) vs. low fear (N = 18) group for the contrast *SPIDERS > ANIMALS*. Significant clusters of the high fear group are shown in purple, significant clusters of the low fear group in blue. Overlapping activation is shown in yellow.

**Fig 4:**
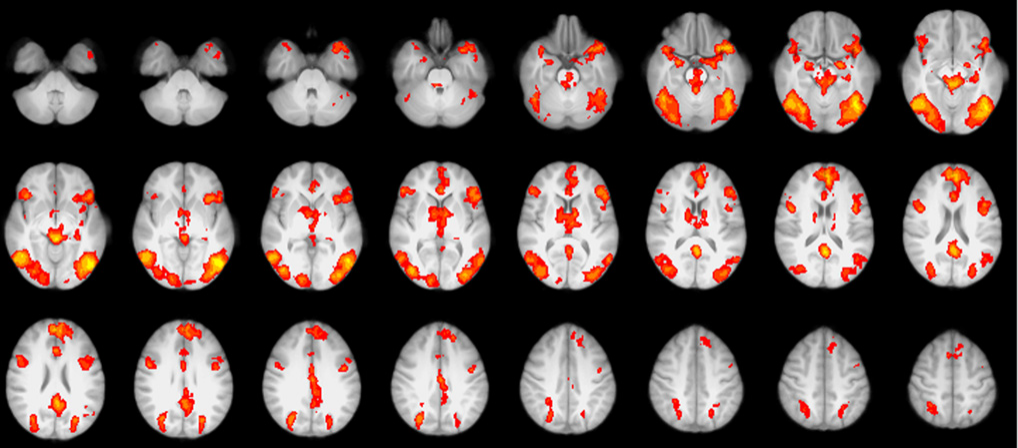
Fear-unspecific contrast. Group effect for the contrast *IAPSnegative > IAPSneutral* (N = 37).

The whole-brain analysis of the fear provocation paradigm showed significant BOLD responses in the orbitofrontal and cingulate cortex, posterior temporal and occipital regions, bilateral anterior insula, medial and lateral prefrontal cortices and cerebellum (see Table 2 and Fig. 3) for the *SPIDERS > ANIMALS* contrast. The contrast *IAPSnegative > IAPSneutral* resulted in significant BOLD responses in the orbitofrontal and cingulate cortex, left insula, bilateral amygdala, and posterior temporal and occipital regions (see Table 3 and Fig. 4). Furthermore, the exploratory whole brain analysis revealed stronger BOLD responses for participants in the high fear group in the cerebellum and anterior cingulate for the contrast *SPIDERS > ANIMA*LS, and stronger BOLD responses for participants in the low fear group in the posterior cingulate for the contrast *IAPSnegative > IAPSneutr*al (see Table 3). Further exploration revealed that the BOLD responses in the posterior cingulate cluster are negative BOLD responses (greater activation during rest than during the task) for both IAPS conditions.

**Table 2:** Higher level whole-brain analysis for the contrast *SPIDERS > ANIMALS*.

Group mean (N = 37)			
Cluster size	Peak (x,y,z)	Peak Z	Location
12117	-6, -56, 24	4.51	Cingulate, frontal gyrus
2297	-58, -40, 24	4.31	Temporal-occipital
2270	28, -78, -38	3.89	Cerebellum
1448	58, -40, 28	3.76	Angular, temporal gyrus
727	58, 10, -4	4.22	Right OFC, insula
582	-50, 8, -10	3.95	Left OFC, insula
**High fear (N = 19)**			
**Cluster size**	**Peak (x,y,z)**	**Peak Z**	**Location**
5487	-2, 26, 28	4.29	ACC
3104	-34, -62, -30	4.1	Cerebellum
1955	-2, -40, 20	3.97	Posterior cingulate
1069	46, -44, 6	3.76	Middle temporal gyrus
856	58, 10, -4	4.18	Right insula
779	-46, 12, -6	4.52	Left insula
753	-58, -42, 26	4.15	Left supramarginal
**Low fear (N = 18)**			
**Cluster size**	**Peak (x,y,z)**	**Peak Z**	**Location**
2762	0, 66, 24	3.78	Frontal pole
2025	-10, -52, 2	4.28	Posterior cingulate
1756	-54, -58, 38	4.08	Left supramarginal
406	52, -68, 30	3.49	Right lateral occipital
379	54, -16, -16	3.50	Right middle temporal
**High fear (N = 19) > low fear (N = 18)**			
**Cluster size**	**Peak (x,y,z)**	**Peak Z**	**Location**
3527	-20, -76, -28	3.49	Cerebellum
454	0, 0, 34	3.59	Anterior cingulate
391	-44, 12, -6	3.74	Left anterior insula
**Low fear (N = 18) > high fear (N = 19)**			
No clusters			

Results are presented for the whole group, both groups separately, and group comparisons.

**Table 3:** Higher level whole-brain analysis for the contrast *IAPSnegative > IAPSneutral*.

Group mean (N = 37)			
Cluster size	Peak (x,y,z)	Peak Z	Location
12350	-50, -68, -4	5.53	Bilateral occipital/temporal
4879	0, -34, -10	5.12	Brain stem and thalamus
3740	-44, 18, -20	4.63	Left insula and OFC
3334	-4, 56, 18	4.72	Cingulate and frontal gyrus
1272	26, -4, -20	5.53	Right amygdala
520	46, 6, 16	4.08	Left amygdala
**High fear (N = 19)**			
**Cluster size**	**Peak (x,y,z)**	**Peak Z**	**Location**
3116	-50, -68, -4	4.45	Left occipital
2826	42, -76, 2	4.38	Right occipital
1070	-44, 16, -18	4.4	Left insula and OFC
578	-6, 52, 18	3.84	ACC
498	-2, -32, -8	4.52	Brain stem
**Low fear (N = 18)**			
**Cluster size**	**Peak (x,y,z)**	**Peak Z**	**Location**
5589	-50, -68, -4	4.45	Left occipital
4836	42, -76, 2	4.38	Right occipital
4537	-44, 16, -18	4.4	Left insula and OFC
2859	-6, 52, 18	3.84	ACC
2790	-2, -32, -8	4.52	Brain stem
416	52, 32, 8	3.52	Right insula and OFC
**High fear (N = 19) > low fear (N = 18)**			
No clusters			
**Low fear (N = 18) > high fear (N = 19)**			
Cluster size	Peak (x,y,z)	Peak Z	Location
374	8, -52, 24	3.48	Posterior cingulate

Results are presented for the whole group, both groups separately, and group comparisons.

A voxelwise analysis restricted to the area of the brain covered by the insular voxel used in the spectroscopy acquisition showed significant BOLD responses for both the *SPIDERS > ANIMALS* and the *IAPSnegative > IAPSneutral* contrast. High and low fear groups only differed for the former contrast, with highly fearful participants showing increased BOLD in the insular spectroscopy voxel compared to low fearful participants (see Table 4). Based on these clusters, functional ROIs within the insula were defined: 1) a fear-specific ROI using the whole-group cluster of the contrast *SPIDERS > ANIMAL*S, 2) a fear-unspecific ROI using the whole-group cluster contrast *IAPSnegative > IAPSneutra*l.

There was no significant difference between the GABA+ levels of low and high fearful participants (*t*[27] = -0.29, *p* = .78).

**Table 4:** Voxel-wise group level analysis, restricted to the regions covered by the spectroscopy voxel.

Contrast *SPIDERS > ANIMALS*			
Group	Cluster size	Peak (x,y,z)	Peak Z
All (N = 37)	121	-48, 14, -8	3.73
High fear (N = 19)	244	-46, 12, -6	4.52
Low fear (N = 18)	No clusters		
High fear > low fear	175	-44, 12, -6	3.74
Low fear > high fear	No clusters		
**Contrast *IAPSnegative > IAPSneutral***			
**Group**	**Cluster size**	**Peak (x,y,z)**	**Peak Z**
All (N = 37)	307	-46, 12, -16	3.91
High fear (N = 19)	97	-34, 18, -2	3.24
Low fear (N = 18)	No clusters		
High fear > low fear	No clusters		
Low fear > high fear	No clusters		

Significant clusters are presented for the two regarded contrast.

### 3.3. The relationship between GABA+ and fear-related BOLD responses

GABA+ concentration did not correlate with % signal change from either the *SPIDERS > ANIMALS* in the fear-specific ROI (all: *r*[23] = .26, *ns*; high fearfulness group: *r*[11] = .39, *ns*; low fearfulness group: *r*[12] = .31, *ns*), or for the contrast *IAPSnegative > IAPSneutral* in the fear-unspecific ROI (all: *r*[24] = -.01, *ns*; high fearfulness group: *r*[11] = -.31, *ns*; low fearfulness group: *r*[12] = .16, *ns*).

**Fig 5:**
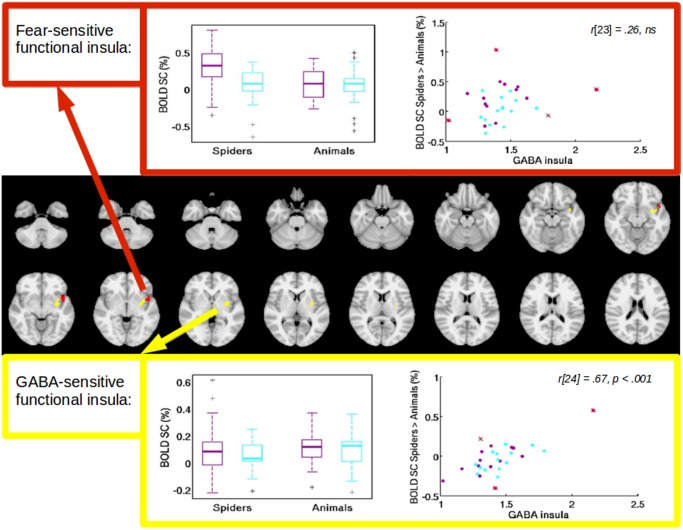
Two clusters within the spectroscopy voxel, both from contrast *SPIDERS > ANIMALS*, red: cluster in the left anterior insula obtained from group level analysis of the contrast *SPIDERS > ANIMALS*, yellow: cluster in the left anterior insula obtained from entering GABA+ as a regressor in the group level analysis. Median %SC from clusters were extracted. ANOVAs were calculated for both ROIs, with a significant interaction for the group-sensitive ROI, and no significant results for the GABA+-sensitive ROI. Purple dots represent participants from the high fear group, blue dots participants from the low fear group, and dots with a strike through participants that were identified as outliers and excluded for correlation. Correlations without outlier exclusion are *r*[27] = .22 (*p* = .24) for the cluster obtained from group level analysis, and *r*[27] = .71 (*p* <. 0001) for the cluster obtained from entering GABA+ as a regressor in the group level analysis.

In order to further investigate the correlation of GABA+ and fear-related BOLD responses, we entered the GABA+ values as a regressor of interest in the whole-brain group level analysis of the fear provocation paradigm. For the contrast *SPIDERS > ANIMALS*, GABA+ predicted BOLD responses in a cluster covering parts of the left amygdala, insula, and ventral striatum (coordinates [x,y,z] = -28,-4,-12, cluster size = 420; see Fig. 5) ), and in the frontal cortex (coordinates [x,y,z] = -46, -26, 58, cluster size = 649). No correlations between GABA+ and BOLD were found for the contrast *IAPSnegative > IAPSneutral*.

## Discussion

The goals of the present study were to investigate BOLD reactivity in the anterior insula during fear provocation, and the role of insular GABA+ concentration and fearfulness in this reactivity. We found increased BOLD responses during fear provocation, this being greater in individuals with high, relative to low, fearfulness. GABA+ concentration in the insular cortex did not differ between the fearfulness groups, and was not associated with BOLD responses in the insula cluster detected during the task. Finally, GABA+ concentration predicted BOLD responses during the task in a different cluster that included brain areas typically associated with the experience of fear, among them part of the insula.

### 4.1. The relationship between fearfulness and neuroimaging measures (BOLD and GABA+) in the insular cortex

Our fear provocation paradigm was designed to elicit fear-specific (pictures of spiders vs. control animals) and fear-unspecific (negative vs. neutral IAPS pictures) BOLD responses. In line with previous results [9,53,54], our paradigm succeeded in eliciting BOLD responses in the anterior insula during both fear-specific and fear-unspecific conditions, along with responses in cingulate cortex, cerebellum and regions within the frontal, temporal and occipital cortices; only the fear-unspecific images brought significant responses in the amygdala, though. Group differences were only observed for the fear-specific BOLD responses, with high fearful participants showing greater responses in cerebellum, anterior cingulate and left anterior insula. These results suggest that fearfulness only influences fear-specific, but not fear-unspecific, BOLD responses. This effect is also reflected at a behavioural level, since we only found group differences in the rating of spider pictures but not negative IAPS pictures. This finding somehow opposes previous results suggesting general increased responses in amygdala and insula to negative stimulation in anxiety prone participants [12] or in phobic participants to general negative—opposite to phobia related—stimulation [8]. However, the two groups in our sample differed more markedly in their scores to the SPQ than to the FSS-II, which could explain why group differences were also more substantial regarding the fear-specific than fear-unspecific stimulation. In any case, whether brain responses to fear related stimuli are qualitatively similar to responses to generally negative stimulation remains unresolved and would require further investigation.

We did, however, find stronger BOLD signals for the low fear group than for the high fear group in the posterior cingulate. The posterior cingulate is often investigated in the context of the default-mode and task-negative network [55,56]. Task-related deactivation of this area was also the case in this study. The group difference we found was caused by less deactivation during presentation of negative vs. neutral IAPS pictures, in participants with low fearfulness only. We speculate that this might indicate that low fearful participants are using an emotion regulation strategy that involves upregulating their default mode network—Goldin et al. [57] found the BOLD in the posterior cingulate associated with reappraisal—however, this needs further investigation.

Previous studies using clinical samples, such as patients with post-traumatic stress disorder, have shown a GABA+ deficit in highly fearful participants over a number of brain regions [13–15]; however, we did not find this to be the case in our non-clinical sample when looking at GABA+ in the insular cortex. It is possible that reduced GABA+ levels are only a marker in clinical populations; although it could also be possible that lower GABA+ concentration is a clinical consequence of the disorder rather than a premorbid factor. Additionally, in some of the previous studies investigated patients were medicated, which could contribute to the measured GABA+ concentration. It also has to be mentioned that not all previous studies report a decrease in GABA+ in highly anxious individuals [58], and whether a decrease can be found might depend on the investigated brain area. In any case, our results suggest no association between GABA+ concentration in the insula and individual differences in fearfulness.

### 4.2. The relationship between GABA+ and fear-related BOLD signals

Even though the %BOLD signal change obtained in the anterior insula in response to fear-specific stimuli did not correlate with GABA+ concentration in the same area of the brain, entering GABA+ as a regressor of interest in the analysis of the fear provocation paradigm revealed a second cluster covering parts of the insula, amygdala and striatum. Unlike most previous studies, though, we found a positive rather than a negative correlation between BOLD and GABA+ [20–23]. This difference in the direction of the relationship could be explained by several factors: A) the voxel from which GABA+ concentration was extracted. Even though interneurons and inhibition are present throughout the cortex, their importance and role might differ from region to region. To our knowledge, there is only one previous study investigating BOLD-GABA+ correlation in the insula [59]. Like the present results, this study reported a positive correlation between both measures, which could indicate that GABA-BOLD relationship depends on the brain area investigated. B) our BOLD measure results from the contrast between two active conditions rather than the comparison to baseline. We did match the stimuli in our conditions with regard to a number of features that could influence the BOLD response, in order to be left with the emotional aspect when contrasting the conditions. Even though there are non-negligible problems with setting up contrasts [60], BOLD responses compared to baseline could be contaminated by factors such as level of visual processing (e.g. how much attention to detail do participants pay), strategies to solve the simple task, and also more physiological factors such as vascular reactivity—factors that should play a reduced role for a contrast. This in mind, previous studies did show a negative correlation between GABA+ and BOLD, but the factors driving that correlation are not yet resolved.

To test whether looking at the contrast vs. main effect makes a difference with regard to the effect of GABA, we calculated the correlation between GABA+ and BOLD for all four main effects (contrast to fixation cross baseline). It turned out that there are no correlations in any but the *ANIMAL* condition, and the correlation is negative (see S1 Table). This suggests that the correlation between BOLD and GABA+ depends on the nature of the BOLD signal investigated. The BOLD signal in the anterior insula, which was found to be influenced by levels of fearfulness, was not influenced by GABA+ levels. The BOLD signal in the cluster sensitive to GABA+ was not influenced by fearfulness, and GABA+ was only associated with BOLD signal during the presentation of animal pictures but not fear-inducing spider pictures or IAPS images. It is possible that GABA related processing plays different roles dependent on stimuli, and that the BOLD signal during the presentation of negatively valenced (spiders and negative IAPS) or complex (IAPS pictures as compared to the pictures of animals) is more strongly influenced by other factors than GABA, which could explain why we only found the GABA-BOLD relationship for the control animal condition. The previous studies that demonstrated the negative correlation between GABA and BOLD used simple visual stimulus material without complex or emotional content to be processed [20–22], except for Northoff et al. [23] using emotional faces, but looking at negative BOLD. The pictures of our control animals are also rather simple stimuli. These results suggest that the GABA concentration in a region might have a specific role in fear-processing in that region. However, in contrast to studies which reported a decrease in fear-specific BOLD responses upon manipulation of GABA transmission [17,18], our study looked at between-subject correlations rather than at within-subject effects. This means, that even though we could not demonstrate a fear-specific relationship between GABA and BOLD, manipulating GABA transmission might still affect the fear-related BOLD responses in the insula we detected. Importantly, the correlation we found between GABA+ and BOLD responses in the control condition of our task indicates that future studies adopting pharmacological manipulation should also investigate the effect of GABAergic drugs on BOLD responses in the control condition of their task.

### Limitations and future directions

Designing our paradigm, we aimed for two contrasts comparing the BOLD signal during the processing of negatively valenced images to the BOLD signal upon neutral stimuli. Participants were asked to rate the images after the scanning session, and as expected negative IAPS pictures were rated as significantly more negative than neutral IAPS pictures, while spider pictures were rated as significantly more negative than pictures of other animals. However, both neutral conditions (neutral IAPS pictures and control animal pictures) were rated as slightly positive with a median of around 7 on a scale from 1–9. Even though this indicates that our neutral conditions were in fact slightly positive conditions, we argue that the positive ratings are result of a contrast effect. In other words, because we did not include positive stimuli in the paradigm, participants might have tried to make use of the whole rating scale which resulted in positive ratings for the neutral stimuli. We did select the control stimuli based on ratings in a previous study [34] and on a pilot we conducted, suggesting that the pictures were in fact neutral. To make sure neutral pictures are actually perceived as neutral, for future studies, it might therefore be an advantage to include positive stimuli, irrespective of the research question.

Our results suggest that a low GABA concentration in the insula is not—as hypothesized—a predisposition for fearfulness and fear-related BOLD responses. This does not necessarily challenge the importance of GABA neurotransmission in fear learning and expression, as frequently demonstrated in animal studies [61]. Even though a negative correlation between GABA concentration in a region and BOLD responses in the same region seems intuitive, the idea of “more GABA, more inhibition, less BOLD” is probably oversimplified. It is in fact not well understood what role GABA and GABAergic interneurons play in the generation of the BOLD signal [62–64], the current understanding being that even though postsynaptic activity in excitatory synapses but not in inhibitory synapses directly influence BOLD, it is the balanced proportional changes in excitation-inhibition activity that lead to the generation of the BOLD response [64]. So even though the negative correlation between GABA+ and BOLD responses in the *ANIMAL* control condition suggests that higher GABA+ levels are associated with higher levels of inhibition and lower BOLD responses, in the future, analysis of GABA as well as excitatory neurotransmitters, such as glutamate, might provide a more complete picture of the relationship between neurochemistry and BOLD responses.

One limitation of this study, common to all research using MRS, is the unspecificity of the MRS signal with regard to the region of interest, but also to the origin of the signal. Due to low signal-to-noise ratio, MRS has very low spatial resolution as compared to the fMRI sequence. The insula voxel was 25 x 30 x 40 mm, and covered areas surrounding the insula, such as frontal areas and the putamen. Furthermore, spatial overlap of the spectroscopy voxel between participants is not exact. This means, we acquired GABA+ concentration from slightly different regions in each participant. Due to increased noise in the insula spectroscopy voxel as compared to other voxel locations, we acquired two spectra from each participant, and excluded bad quality spectra based on expert ratings. The amount of spectra that were excluded reflects the challenge related to obtaining GABA+ data from more noisy regions, and the data loss is a limitation of this study. Given the restricted data quality, further studies are needed to support our results since it is possible that the positive correlation we found is a false positive result.

Furthermore, within the measured regions, the origins of the GABA+ signal are not clear. The concentration measure is unspecific to whether GABA is intra- or extracellular, so it does not necessarily give an indication of GABA transmission. Low GABA concentration could either indicate a less established interneuron network, with less interneurons, or less connected interneurons. On the other hand, GABA concentration could be a state marker of interneuronal activity or current GABA availability. Last but not least, the GABA+ peak in the spectrum is influenced by macromolecules [65] however, studies such as this investigating links between behaviour and GABA have been proposed to be less influenced by the macromolecule signal than those comparing GABA+ levels patients and control groups [66].

We cannot rule out that the fear-inducing paradigm had an effect on the GABA+ concentration, potentially influencing the correlation between BOLD responses and GABA+ concentration. Recently, a few studies have suggested that GABA+ may be subject to changes induced by experimental manipulation, such as stress induction [67]. Also the activation of a brain region can have an influence on later measured GABA+ levels, as shown by Michels et al. [68]. These findings indicate that GABA+ levels are not completely stable. In our scanning protocol, we performed the GABA+ spectroscopy at the end of the session. We cannot rule out that the functional paradigm or potentially even the scanning situation itself altered the GABA+ levels in our participants.

The sample used in this study exclusively consisted of female participants. This somewhat limits the generalizability of the findings. However, investigating female populations is particularly important in the face of higher prevalence of anxiety disorders in females as well as stronger BOLD responses to negatively valenced stimuli [69]. Since gender differences in the GABA-BOLD correlation cannot be excluded, replication of our findings in a male sample would be required.

### Conclusions

We found fear-related BOLD responses brain regions that have been previously associated with emotion processing. BOLD responses in the insula were stronger for participants with high fear than for participants with low fear, but we did not find any group differences in GABA+ concentration. We found a positive correlation between fear-related BOLD and GABA+ concentration in a cluster including insula, putamen and amygdala. This was in contrast to our expectations, and suggests that whether a positive vs. negative relationship between GABA+ and BOLD is found depends on the investigated region and the nature of the contrast.

## Supporting Information

S1 Fig All spectra that were included in the analysis (48/74) are plotted in the frequency range 2–4 ppm. The GABA+ peak can be found at 3 ppm.
(PNG)

S1 File Establishment of the emotion paradigm. (PDF)

S1 Table GABA+ BOLD correlations. Correlations between GABA+ and BOLD signal changes for contrasts and main effects (contrast to fixation cross).
(DOC)
