# Supplementary figures and images for: The Relationship between Fearfulness, GABA+, and Fear-Related BOLD Responses in the Insula
Source: PLoS One. 2015 Mar 26;10(3):e0120101. doi: 10.1371/journal.pone.0120101 (PMC4374765; doi:10.1371/journal.pone.0120101)

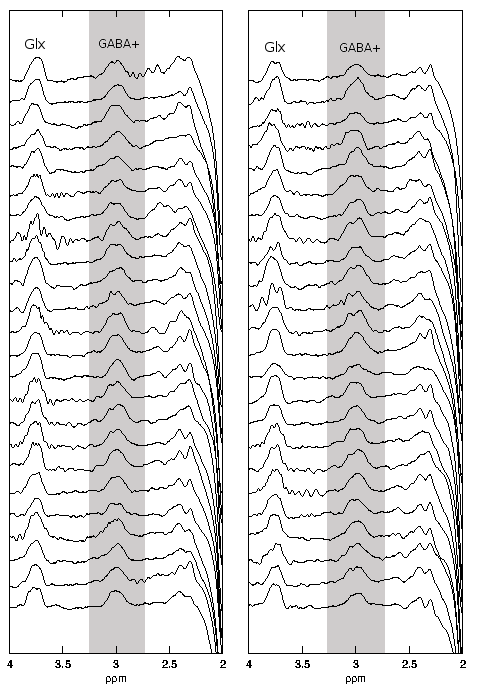

Supplement: S1 Fig — The GABA+ peak can be found at 3 ppm. (PNG) [file pone.0120101.s001.png]
