# Supplementary material for: The Relationship between Fearfulness, GABA+, and Fear-Related BOLD Responses in the Insula
Source: PLoS One. 2015 Mar 26;10(3):e0120101. doi: 10.1371/journal.pone.0120101 (PMC4374765; doi:10.1371/journal.pone.0120101)
Supplement: S1 File — (PDF) [file pone.0120101.s002.pdf]

Establishment of the emotion paradigm

The emotion paradigm that was presented during fMRI was designed to 1) elicit fear reactions by showing fear-sensitive stimuli (pictures of spiders) as compared to control stimuli and 2) elicit more general negative emotion processing (unspecific negative pictures chosen from the International Affective Picture System (Lang et al., 2008) as compared to neutral pictures).

The stimuli fall into four categories: negative IAPS pictures, neutral IAPS pictures, spider pictures, and other animal pictures (neutral control). Two control conditions (neutral IAPS and other animals) were chosen in order to make fair contrasts (*IAPSnegative* > *IAPSneutral*, *SPIDERS* > *ANIMALS*) where the two conditions were designed in a way that they did not differ in any possible confounding factor other than their emotional/fear content. Confounding factors controlled for are: human content (Colden et al., 2008), brightness, contrast or spatial frequency (Carretié et al., 2007; Said et al., 2008; Vlamings et al., 2009; Vuilleumier et al., 2001), and complexity (Wiens et al., 2011). Spatial frequency was calculated for eight frequency bands and three colours with the scripts provided by Delplanque et al. (2007). Brightness was calculated as the average of pixels' mean RGB (red/green/blue) values. Contrast scores were obtained by extracting the standard deviation of pixels' mean RGB values in each pixel column and then computing the standard deviation of these values (also see Bradley et al., 2007, Ihssen & Keil, 2012). Complexity was rated by the researcher on a scale of 1 to 10.

IAPS pictures were selected according to the valence reported in Lang et al. (2008). Negative IAPS pictures had valence ratings between 1 and 3 (mean = 1.9), neutral IAPS pictures had valence ratings between 4 and 6.5 (mean = 4.9). They were matched for content by broadly classifying the pictures into four categories and including 10 pictures of each category in the stimulus selection. The categories were: human bodies (definition: main aspect of picture is a human body), human faces (definition: human face looking at camera), animals, and environment (environmental situation, bodies - if visible - were not main aspect of image). They did not differ in format (negative: 8 portrait, neutral 6 portrait), brightness (negative mean = 98 [std. = 36], neutral mean = 85 [std. = 34], z = 1.6 [p =.10]) or contrast (negative mean = 17 [std. = 9], neutral mean = 17 [std. = 8], z = -0.43 [p = .67]), or in any of the eight frequency bands for red green or blue.

Picture numbers IAPS negative: 1052, 1111, 1525, 1932, 9140, 9181, 9183, 9185, 9187, 9571, 3001, 3005, 3015, 3063, 3140, 6825, 9040, 9362, 9412, 9413, 2095, 2800, 3059, 3168, 3180, 3266, 3301, 6231, 6250, 6563, 5971, 9280, 9611, 9620, 9622, 9630, 9908, 9911, 9930, 9940

Picture numbers IAPS neutral: 1112, 1350, 1390, 1616, 1661, 1670, 1675, 1726, 1820, 1908, 2272, 2357, 2377, 2396, 2400, 2410, 2411, 2480, 2749, 9210, 2190, 2210, 2215, 2221, 2230, 2440, 2441, 2495, 2499, 6837, 7130, 7180, 7242, 7491, 7500, 7510, 7546, 7560, 7595, 8211

Spider images and images of the control animals were selected from the internet. For the control images, images of six categories butterflies, lady bugs, birds, lizards, snails and caterpillars were selected and presented to 20 female raters in line with a selection of spider images. Images showed the animal in different environments, in half of the finally selected stimuli, human hands were visible touching or in close distance to the animal. From the six preselected categories, four were selected based on the mean valence ratings for the images. The most neutrally rated categories were birds (mean valence = 6.4), lizards (mean valence = 5.2), caterpillars (mean valence = 4.7) and snails (mean valence = 4.4; overall mean = 5.2). As expected, the images of spiders were rated with a mean negative valence of 2.6. The selected images for spiders did not significantly differ from the control animal images in format (all portrait), brightness (spiders mean = 117 [std. = 24], neutral mean = 124 [std. = 30], z = -0.88, p = .38) or contrast (mean spiders = 11 [4], neutrals = 10 [4], z = 0.60, p = .55) or in any of the eight frequency bands for red green or blue. Complexity

was assumed to be balanced by the fact that all images showed an animal on a background.

The images were presented in short blocks of 10 seconds, with 4 images (presented for 2.5 sec.) each. A block design was chosen because block designs increase power to detect activation, the duration of 10 seconds was chosen to minimalize habituation effects within the blocks. In order to keep attention to the pictures and also to decrease habituation, the 4 images presented came from different categories (different IAPS categories, or different control animals) except for the spider blocks that exclusively showed spiders. In the animal blocks either all or none of the images within a block showed human hands.

The blocks were arranged in superblocks (consisting of one block of each kind) whereby the blocks within the superblocks were pseudorandomized. Then, the superblocks were pseudorandomized. This assured that blocks of one kind could occur subsequently maximal twice in a row. After half of the blocks a fixation cross appeared for either 7, 9, 11 or 13 seconds (there were no blocks of the same kind subsequently without a fixation period in between).

References

Colden, A., Bruder, M., Manstead, A.S.R., 2008. Human content in affect-inducing stimuli: A secondary analysis of the international affective picture system. Motivation and Emotion 32, 260–269.

Carretié, L., Hinojosa, J.A., López-Martín, S., Tapia, M., 2007. An electrophysiological study on the interaction between emotional content and spatial frequency of visual stimuli. Neuropsychologia 45, 1187–1195.

Said, C.P., Baron, S.G., Todorov, A., 2008. Nonlinear amygdala response to face trustworthiness: contributions of high and low spatial frequency information. Journal of Cognitive Neuroscience 21, 519–528.

Vlamings, P., Goffaux, V., Kemner, C., 2009. Is the early modulation of brain activity by fearful facial expressions primarily mediated by coarse low spatial frequency information? Journal of Vision 9, 1–13.

Vuilleumier, P., Armony, J.L., Driver, J., Dolan, R.J., 2001. Effects of attention and emotion on face processing in the human brain: an event-related fMRI study. Neuron 30, 829–841.

Wiens, S., Sand, A., Olofsson, J.K., 2011. Nonemotional features suppress early and enhance late emotional electrocortical responses to negative pictures. Biological Psychology 86, 83–89.

Delplanque, S., N’diaye, K., Scherer, K., Grandjean, D., 2007. Spatial frequencies or emotional effects? A systematic measure of spatial frequencies for IAPS pictures by a discrete wavelet analysis. Journal of Neuroscience Methods 165, 144–50.

Bradley, M.M., Hamby, S., Löw, A., Lang, P.J., 2007. Brain potentials in perception: picture complexity and emotional arousal. Psychophysiology 44, 364–73.

Ihssen, N., Keil, A., 2012. Accelerative and decelerative effects of hedonic valence and emotional arousal during visual scene processing. Q J Exp Psych 66, 1276–301.

Lang, P., Bradley, M., Cuthbert, B., 2008. International affective picture system (IAPS): Affective ratings of pictures and instruction manual. Technical Report A-8. University of Florida, Gainesville, FL.
