# Supplementary material for: The Relationship between Fearfulness, GABA+, and Fear-Related BOLD Responses in the Insula
Source: PLoS One. 2015 Mar 26;10(3):e0120101. doi: 10.1371/journal.pone.0120101 (PMC4374765; doi:10.1371/journal.pone.0120101)
Supplement: S1 Table — Correlations between GABA+ and BOLD signal changes for contrasts and main effects (contrast to fixation cross). (DOC) [file pone.0120101.s003.doc]

Table S1: GABA+ BOLD correlations. Correlations between GABA+ and BOLD signal changes for contrasts and main effects (contrast to fixation cross).

| ROI | *IAPS contrast* | *SPIDERS > ANIMALS* | *IAPSnegative* | *IAPSneutral* | *SPIDERS* | *ANIMALS* |
| --- | --- | --- | --- | --- | --- | --- |
| Fear-specific | *r*[23] = .05, *ns* | *r*[23] = .26, *ns* | *r*[25] = .32, *ns* | *r[*23] = -.37, *ns* | *r*[23] = .10, *ns* | *r*[24] = -.13, *ns* |
| GABA-sensitive | *r*[24] = .19, *ns* | *r*[24] = .67, *p* < .001 | *r*[26] = .09, *ns* | *r*[24] = -.13, *ns* | *r*[23] = .03, *ns* | *r*[24] = -.74, *p* < .0001 |
| Fear-unspecific | *r*[24] = -.01, *ns* | *r*[24] = .42, *p* = .03 | *r*[23] = -.10, *ns* | *r*[24] = .00, *ns* | *r*[24] = -.02, *ns* | *r*[25] = -.30, *ns* |
